# Trunk‐to‐Appendicular Fat Ratio and Blood Pressure: Survey‐Weighted Regression, Spline Modeling, and Exploratory Lipid Attenuation Analysis

**DOI:** 10.1155/ijhy/3281602

**Published:** 2026-07-18

**Authors:** Bao The Nguyen, Tien Anh Hoang

**Affiliations:** ^1^ Department of Internal Medicine, University of Medicine and Pharmacy, Hue University, Hue, Vietnam, hueuni.edu.vn

**Keywords:** blood pressure, body composition, dual-energy X-ray absorptiometry (DXA), National Health and Nutrition Examination Survey (NHANES), trunk-to-appendicular fat ratio (TAR)

## Abstract

Central adiposity is closely linked to blood pressure (BP), yet body mass index (BMI) and waist circumference (WC) may not fully capture fat distribution. We analyzed 4307 adults from NHANES 2011–2018 with whole‐body dual‐energy X‐ray absorptiometry (DXA) to examine whether the trunk‐to‐appendicular fat ratio (TAR), a marker of trunk‐predominant adiposity, was associated with BP. Survey‐weighted multivariable linear regression and restricted cubic spline models were applied. TAR showed the strongest correlation with systolic BP (SBP) and diastolic BP (DBP) (*r* = 0.326 and 0.330, respectively; both *p* < 0.001). After adjustment for demographic and clinical covariates and either BMI or WC, TAR remained associated with higher SBP and DBP (*p* < 0.001). Additional adjustment for serum lipids modestly attenuated these associations, with triglycerides showing the largest attenuation. Higher TAR was consistently associated with higher predicted BP, supporting TAR as a useful adiposity index for hypertension research.

## 1. Introduction

Hypertension is a pervasive global health challenge and a leading cause of premature cardiovascular mortality. An estimated 1.3 billion adults worldwide—roughly one in every five—suffer from elevated blood pressure (BP) [1]. Each year, hypertension is responsible for approximately 9.4 million deaths globally [2], accounting for about 45% of heart disease and 51% of stroke fatalities [3]. This growing burden of hypertension underscores the urgent need to better characterize modifiable determinants and early phenotypic markers to improve prevention efforts.

Excess adiposity—particularly central fat accumulation—has been a key driver in the rising prevalence of hypertension. Epidemiological studies have shown a strong association between central fat accumulation and elevated BP. For instance, individuals with an enlarged waist circumference (WC) have roughly double the odds of developing hypertension compared to those with a normal waist size [1]. Notably, individuals with normal body weight may still face an elevated risk when they exhibit a disproportionate accumulation of abdominal fat, a phenotype termed normal‐weight central obesity that confers a risk of hypertension comparable to or even exceeding that observed in overweight individuals without central fat concentration [4]. These findings suggest that, beyond the overall degree of adiposity, the pattern of fat distribution is also an important dimension of cardiovascular risk.

While body mass index (BMI) is widely used to define overweight/obesity, this conventional metric does not capture fat distribution. A substantial body of evidence suggests that measures of central or shape‐related adiposity, including WC, waist‐to‐height ratio (WHtR), and body roundness index (BRI), may better characterize hypertension and cardiometabolic risk than BMI alone in some populations [4]. Additionally, advances in body composition imaging, particularly dual‐energy X‐ray absorptiometry (DXA), now enable the direct quantification of regional fat deposits with high accuracy. DXA allows separate estimation of trunk and appendicular fat mass, providing a detailed analysis of fat distribution across the trunk and limbs [5]. Using DXA, researchers can define novel indices that reflect fat distribution rather than total body size alone. One such metric—the trunk‐to‐appendicular fat ratio (TAR)—is calculated as trunk fat mass divided by appendicular fat mass (sum of arm and leg fat). TAR represents the predominance of central adiposity relative to peripheral fat stores, thereby capturing central‐to‐peripheral fat patterning. Rather than serving as a direct measure of visceral fat, TAR may provide a useful proxy of relative central fat accumulation [6]. Prior studies demonstrate that DXA‐derived TAR is a more precise indicator of abdominal fat distribution than WHR [6]. This index has been shown to better reflect metabolic disturbances associated with abdominal obesity than traditional anthropometric ratios in some settings. Central adiposity is also closely linked to atherogenic dyslipidemia, including higher triglyceride‐rich lipoproteins, higher LDL‐cholesterol, and lower HDL‐cholesterol [7]. Dyslipidemia may contribute to BP elevation through shared vascular pathways, particularly endothelial dysfunction, oxidative stress, impaired vascular tone, arterial stiffness, and vascular remodeling [8, 9]. Therefore, if the association between trunk‐predominant adiposity and BP were largely explained by lipid abnormalities, adjustment for standard lipid parameters would be expected to attenuate the TAR–BP association. However, obesity‐related hypertension is multifactorial and is also driven by nonlipid mechanisms, including sympathetic nervous system activation, renin–angiotensin–aldosterone system activation, renal sodium retention, insulin resistance, adipose inflammation, and leptin‐related neurohormonal pathways [10]. For this reason, lipid‐adjusted models may help clarify whether routinely measured lipid parameters account for part of the TAR–BP association, but they should not be interpreted as formal causal mediation analysis.

However, research on TAR in relation to hypertension in adults remains limited. Most prior studies have focused on anthropometric indicators, such as BMI, WC, WHtR, and BRI, rather than directly examining DXA‐derived fat distribution ratios in relation to BP. Aside from investigations in pediatric or specific subpopulations [11], data on TAR and hypertension in general adult populations are scarce. Moreover, it remains unclear whether TAR captures BP‐related adiposity patterning beyond conventional measures and whether this relationship is partly attenuated by lipid parameters. We leveraged the U.S. National Health and Nutrition Examination Survey (NHANES 2011–2018)—a large, nationally representative dataset—to evaluate the association between TAR and BP in adults. In addition, we explored this relationship using survey‐weighted regression and restricted cubic spline modeling and examined whether adjustment for standard lipid parameters attenuated the TAR–BP association. By focusing on body fat distribution rather than total adiposity alone, this study aims to extend current evidence on adiposity phenotypes relevant to hypertension risk.

## 2. Methods

### 2.1. Setting and Design

We utilized data from the NHANES 2011–2018, a nationally representative, continuous, cross‐sectional survey conducted by the National Center for Health Statistics (NCHS). NHANES employs a complex, multistage, stratified probability sampling design to obtain a sample that is representative of the noninstitutionalized U.S. civilian population. Specific subgroups—including older adults, racial/ethnic minorities, and individuals with lower socioeconomic status—are oversampled to improve the precision of the estimates. Standardized interviews, physical examinations, and laboratory assessments were conducted in participants’ homes and at Mobile Examination Centers (MECs) by trained personnel, following rigorous quality control protocols. Detailed documentation of survey operations and data quality is publicly available on the NHANES website [12].

**FIGURE 1:**
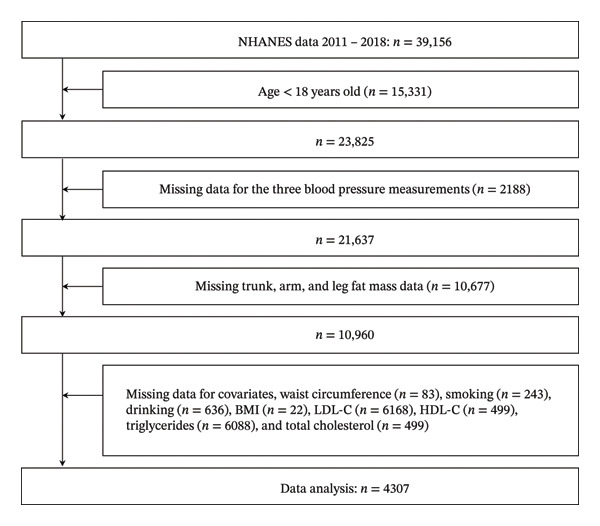
Flowchart of study participants.

Among the initial 39,156 individuals examined between 2011 and 2018, we excluded participants aged < 18 years and those with missing whole‐body DXA‐derived trunk, arm, or leg fat mass; missing values for all three BP measurements; and those with incomplete covariate or lipid data. The final analytic sample included 4307 adults (Figure 1). NHANES protocols were approved by the NCHS Research Ethics Review Board, and all participants provided written informed consent.

### 2.2. BP

BP was measured in the NHANES MEC using a standardized protocol conducted by certified physician examiners. After participants rested quietly in a seated position for at least 5 min, their maximum inflation level was determined, followed by three consecutive BP measurements. If any reading was interrupted or technically inadequate, a fourth attempt was performed. Both systolic BP (SBP) and diastolic BP (DBP) were recorded using the right arm unless a medical condition—such as rashes, dressings, casts, edema, paralysis, arteriovenous shunts, open wounds, or a history of radical mastectomy—necessitated use of the left arm. Prior to measurement, upper‐arm circumference was obtained to ensure appropriate cuff selection based on NHANES guidelines. BP examiners were trained and certified through the Shared Care Research and Education Consulting program, and all measurements were performed under strict quality control procedures. The mean of the three valid readings was used for analysis [12].

### 2.3. TAR

Body fat distribution was assessed using whole‐body DXA. All scans were performed at NHANES MECs using Hologic Discovery/A densitometers and analyzed with Hologic APEX software Version 4.0 (Hologic Inc., Bedford, MA), following standardized NHANES DXA quality control procedures. The DXA system provides region‐specific body composition measures based on predefined anatomical boundaries. For each participant, we obtained trunk fat mass (g), arm fat mass (g), leg fat mass (g), appendicular fat mass (sum of arm and leg fat mass, g), and total body fat percentage (%) [12]. The main exposure variable in this study was the TAR, calculated as trunk fat mass divided by appendicular fat mass. TAR reflects the relative predominance of central (trunk) fat compared with peripheral (limb) fat stores.

### 2.4. Covariates

In addition to the DXA‐derived body fat parameters, a comprehensive set of demographic and clinical covariates was collected to account for potential confounding. These included age, sex, and race/ethnicity (classified according to the NHANES RIDRETH1 categories), as well as anthropometric measures such as BMI and WC. Diabetes status was determined based on self‐reported physician diagnosis (DIQ010). Smoking status was derived from SMQ020 and SMQ040, whereas drinking status was defined using ALQ101 for NHANES 2011–2016 and ALQ121 for NHANES 2017‐2018. Antihypertensive treatment was identified from NHANES prescription medication data and defined as either reported use of antihypertensive medications or self‐reported current treatment for hypertension. Because missingness for the antihypertensive treatment variable exceeded 50%, participants with missing treatment status were retained in the analysis and modeled as a separate category. For analyses involving lipid outcomes or for models assessing the relationship between TAR and BP while considering metabolic factors, serum lipid indices—including HDL‐cholesterol, LDL‐cholesterol, triglycerides, and total cholesterol—were incorporated as additional covariates.

### 2.5. Statistical Analysis

All analyses were performed in R Version 4.5.0 (R Foundation for Statistical Computing, Vienna, Austria) [13]. The complex multistage sampling design of NHANES was incorporated in all primary analyses by applying MEC examination weights, strata, and primary sampling units using the survey package [14]. Baseline characteristics were described as survey‐weighted means with 95% confidence intervals (CIs) for continuous variables and weighted percentages (95% CIs) for categorical variables. Crude relationships between adiposity and BP were explored using survey‐weighted Pearson correlation coefficients for TAR, trunk fat mass, arm fat mass, leg fat mass, total body fat percentage, and both SBP and DBP. Correlation matrices were displayed as correlogram plots, with scatterplots in the lower panels, smoothed distributions on the diagonal, and correlation coefficients (*r*) with significance levels in the upper panels.

Multivariable survey‐weighted linear regression models were fitted to quantify the associations of TAR with SBP and DBP. Because BMI and WC showed high collinearity when entered together, adjusted models were specified separately with either BMI or WC, together with age, sex, race/ethnicity, antihypertensive treatment, smoking, drinking, and diabetes status. Regression coefficients (*β*) and *p* values are reported. The functional form of the TAR–BP association was further examined using restricted cubic spline regression with four knots, implemented in the rms package in separate models for SBP and DBP [15]. From these models, spline curves with 95% CIs were plotted using the ggplot2 package [16], with vertical reference lines at TAR quartiles, and Wald χ^2^ tests from model ANOVA provided *p* values for both the overall association and the nonlinear component of TAR. WHtR was calculated as WC divided by height, and BRI was calculated using the standard anthropometric formula based on WC and height. To compare TAR with WHtR, we performed a survey‐weighted bootstrap comparison of dependent correlations between each adiposity index and BP. We also performed supporting incremental model analyses to determine whether TAR explained additional variance in BP beyond total fat mass. Base models included total fat mass and covariates, and TAR was then added to estimate the change in survey‐weighted *R* ^2^ and the statistical significance of the added TAR term. In additional subgroup analyses, TAR × sex and TAR × race/ethnicity interaction terms were tested in survey‐weighted regression models. When a significant race/ethnicity interaction was observed, race/ethnicity‐specific TAR slopes and selected pairwise comparisons were estimated, with Holm adjustment applied for multiple comparisons. All tests were two‐sided, and *p* < 0.05 was considered statistically significant.

Independent associations of serum lipids with BP were evaluated in separate survey‐weighted linear regression models for each lipid, with SBP or DBP as the dependent variable and the same set of covariates. To explore whether serum lipids accounted for part of the TAR–BP association, we performed an exploratory attenuation analysis. Before fitting the multivariable models, multicollinearity among TAR, BMI, and WC was assessed. TAR showed no evidence of problematic collinearity, whereas BMI and WC were highly collinear when entered simultaneously in the same model (VIFs: TAR = 2.07, BMI = 9.00, and WC = 10.52). Therefore, to reduce multicollinearity, BMI and WC were not included in the same model. Instead, two separate models were constructed: one model adjusted for age, sex, race/ethnicity, hypertension treatment, smoking, drinking, diabetes, and BMI, whereas the other adjusted for age, sex, race/ethnicity, hypertension treatment, smoking, drinking, diabetes, and WC. For each lipid, model (a) included TAR and the covariates without the lipid parameter. Model 1 included age, sex, race/ethnicity, antihypertensive treatment, smoking, drinking, diabetes, and BMI, whereas Model 2 included the same covariates but replaced BMI with WC. Model (b) additionally adjusted for the corresponding lipid parameter. The percentage attenuation of the TAR–BP association was calculated as (*β*_*a*_ − *β*_*b*_)/*β*_*a*_ × 100%, where *β*_*a*_ and *β*_*b*_ are the TAR coefficients from models (a) and (b), respectively [17]. A relative reduction in the TAR coefficient of ≥ 10% was prespecified as a pragmatic change‐in‐estimate threshold, suggesting that the lipid parameter may account for a nonnegligible portion of the TAR–BP association [18, 19].

## 3. Results

A total of 4307 adults who met the eligibility criteria were included in the final analysis. The survey‐weighted results showed a slightly higher proportion of men than women, with men accounting for 52.5% of the study population. Women had higher trunk fat mass, arm fat mass, leg fat mass, and total body fat percentage than men (all *p* < 0.001). However, men exhibited a significantly greater TAR than women (1.11 vs. 0.91; *p* < 0.001) (see Table 1).

**TABLE 1:** Baseline characteristics of participants.

Characteristics	Male (*n* = 2213)	Female (*n* = 2094)	Total (*n* = 4307)	*p*
Age (years)	38.12 (37.37, 38.88)	38.70 (37.92, 39.48)	38.40 (37.83, 38.97)	0.267
BMI	28.14 (27.77, 28.52)	28.64 (28.20, 29.07)	28.38 (28.06, 28.69)	0.057
Waist‐to‐height ratio	0.560 (0.555, 0.565)	0.583 (0.577, 0.590)	0.571 (0.566, 0.576)	< 0.001
Body roundness index	4.69 (4.57, 4.81)	5.27 (5.11, 5.42)	4.97 (4.86, 5.08)	< 0.001
Race				0.002
Mexican American	11.5 (8.9, 14.2)	9.5 (7.3, 11.7)	10.6 (8.3, 12.9)	
Other Hispanic	7.9 (6.0, 9.8)	6.9 (5.0, 8.7)	7.4 (5.7, 9.2)	
Non‐Hispanic White	62.1 (57.9, 66.3)	62.1 (57.8, 66.4)	62.1 (58.2, 66.1)	
Non‐Hispanic Black	9.3 (7.4, 11.3)	11.4 (9.1, 13.8)	10.3 (8.4, 12.3)	
Non‐Hispanic Asian	5.8 (4.7, 7.0)	5.9 (4.7, 7.1)	5.9 (4.8, 7.0)	
Other race	3.3 (2.1, 4.5)	4.1 (2.9, 5.3)	3.7 (2.7, 4.6)	
Trunk fat mass (g)	12205.37 (11830.87, 12579.87)	14026.22 (13623.29, 14429.15)	13070.02 (12763.41, 13376.63)	< 0.001
Arm fat mass (g)	2873.20 (2797.22, 2949.19)	3780.25 (3681.72, 3878.77)	3303.92 (3235.13, 3372.72)	< 0.001
Leg fat mass (g)	8017.32 (7820.27, 8214.38)	11393.52 (11128.42, 11658.63)	9620.55 (9430.62, 9810.48)	< 0.001
Total body fat percentage (%)	27.07 (26.68, 27.47)	38.50 (38.05, 38.96)	32.50 (32.10, 32.90)	< 0.001
Trunk‐to‐appendicular fat ratio	1.11 (1.09, 1.12)	0.91 (0.90, 0.93)	1.02 (1.00, 1.03)	< 0.001
Triglycerides (mg/dL)	120.51 (116.16, 124.87)	99.48 (95.52, 103.43)	110.52 (107.13, 113.92)	< 0.001
Total cholesterol (mg/dL)	186.68 (184.55, 188.81)	190.06 (188.00, 192.12)	188.28 (186.72, 189.84)	0.020
HDL cholesterol (mg/dL)	48.55 (47.81, 49.28)	58.34 (57.27, 59.41)	53.20 (52.45, 53.94)	< 0.001
LDL cholesterol (mg/dL)	114.03 (112.30, 115.76)	111.82 (109.93, 113.71)	112.98 (111.69, 114.27)	0.086
Hypertension	23.8 (21.0, 26.6)	20.1 (18.0, 22.2)	22.0 (20.1, 24.0)	0.013
Diabetes mellitus	5.2 (4.0, 6.3)	5.3 (4.2, 6.5)	5.3 (4.4, 6.1)	0.820
Smoking	47.7 (44.1, 51.3)	33.9 (30.9, 36.8)	41.1 (38.5, 43.8)	< 0.001
Drinking	88.3 (86.5, 90.2)	77.2 (74.2, 80.2)	83.1 (81.1, 85.1)	< 0.001
HTN treatment				< 0.001
Yes	18.9 (16.4, 21.5)	17.3 (15.2, 19.3)	18.2 (16.3, 20.0)	
No	6.7 (5.4, 8.0)	4.0 (3.0, 5.1)	5.4 (4.6, 6.3)	
Missing	74.4 (71.7, 77.1)	78.7 (76.5, 80.9)	76.4 (74.4, 78.5)	

*Note:* All estimates are weighted.

**FIGURE 2:**
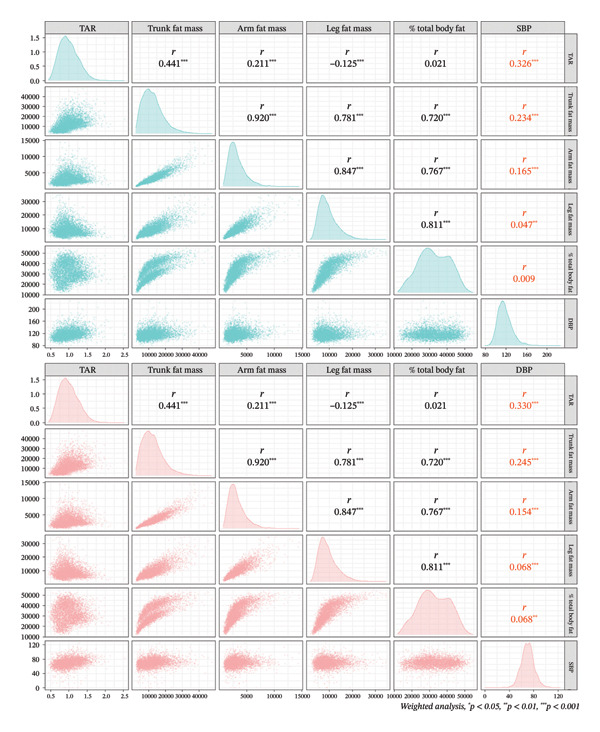
Correlogram illustrating the relationships between the trunk‐to‐appendicular fat ratio, regional and total body fat, and blood pressure.

A positive correlation was identified between the TAR and both SBP (*r* = 0.326, *p* < 0.001) and DBP (*r* = 0.330, *p* < 0.001) (Figure 2). Significantly, the magnitude of these associations exceeded those observed for regional fat depots and total body fat, indicating that TAR demonstrated the strongest correlation with BP among the adiposity measures evaluated. Additionally, to directly compare TAR with WHtR, we performed a survey‐weighted bootstrap comparison of dependent correlations. The correlation between TAR and SBP was significantly stronger than that between WHtR and SBP (*r* = 0.326 vs. 0.269; Δr = 0.058, 95% CI: 0.023–0.092; *p* = 0.001). A similar pattern was observed for DBP, where TAR also showed a stronger correlation than WHtR (*r* = 0.330 vs. 0.233; Δr = 0.097, 95% CI: 0.060–0.134; *p* < 0.001) (Supporting Table 1).

**FIGURE 3:**
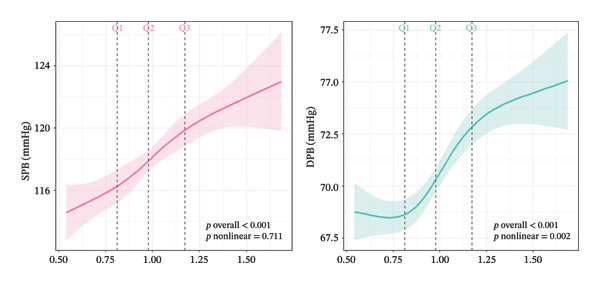
Adjusted restricted cubic spline curve for the association between TAR and blood pressure.

The spline analyses showed significant positive associations between TAR and both SBP and DBP (Figure 3). For SBP, the association was overall significant (*p* overall < 0.001), with no clear evidence of nonlinearity (*p*nonlinear = 0.711), indicating an approximately linear increase across the TAR range. For DBP, the association was also overall significant (*p* overall < 0.001) and showed evidence of nonlinearity (*p* nonlinear = 0.002), with a steeper increase around intermediate TAR values followed by a more gradual rise at higher TAR levels.

In survey‐weighted univariable linear regression, TAR was positively associated with both SBP (*β* = 7.054, *p* < 0.001) and DBP (*β* = 7.246, *p* < 0.001). Among conventional adiposity indicators, BMI was positively associated with SBP but not DBP, whereas WC was not significantly associated with either SBP or DBP. TAR was also associated with lipid parameters, showing inverse association with HDL‐cholesterol and positive associations with LDL‐cholesterol, triglycerides, and total cholesterol (Table 2). Additional supporting analyses were performed to examine the incremental and subgroup‐specific associations of TAR with BP. In models including total fat mass and covariates, adding TAR increased the survey‐weighted *R* ^2^ from 0.224 to 0.236 for SBP and from 0.169 to 0.190 for DBP, corresponding to additional explained variance of 1.16 and 2.07 percentage points, respectively. The added TAR term was statistically significant for both outcomes (both *p* < 0.001; Supporting Table 2). We also tested interaction terms for TAR by sex and race/ethnicity. The TAR×sex interaction was significant for both SBP (*p* = 0.002) and DBP (*p* = 0.031), with positive TAR–BP associations observed in both men and women (Supporting Table S3). The TAR × race/ethnicity interaction was significant for SBP (*p* < 0.001), but not for DBP (*p* = 0.192). Race/ethnicity‐specific TAR–SBP slopes and selected pairwise comparisons are presented in Supporting Tables S4 and S5.

**TABLE 2:** Survey‐weighted univariable associations of trunk‐to‐appendicular fat ratio, anthropometric indices, and covariates with blood pressure and lipid parameters.

*β*	SBP	DBP	HDL‐c	LDL‐c	Triglycerides	TC
TAR	7.054^∗∗∗^	7.246^∗∗∗^	−13.357^∗∗∗^	17.641^∗∗∗^	93.048^∗∗∗^	22.923^∗∗∗^
Age	0.203^∗∗∗^	0.149^∗∗∗^	0.217^∗∗∗^	0.729^∗∗∗^	0.198	0.985^∗∗∗^
Female	−4.517^∗∗∗^	−1.487^∗∗^	6.964^∗∗∗^	1.318	−1.215	8.046^∗∗∗^
BMI	0.426^∗∗∗^	0.025	−0.329^∗∗^	0.126	0.702	−0.064
Waist circumference	−0.059	0.072	−0.137^∗∗^	0.120	0.169	0.018
WHtR, per 0.1 increase	3.973^∗∗∗^	2.698^∗∗∗^	−5.055^∗∗∗^	6.515^∗∗∗^	21.058^∗∗∗^	5.689^∗∗∗^
BRI	1.669^∗∗∗^	1.093^∗∗∗^	−2.082^∗∗∗^	2.486^∗∗∗^	8.600^∗∗∗^	2.131^∗∗∗^
Race						
Mexican American	1 (ref)	1 (ref)	1 (ref)	1 (ref)	1 (ref)	1 (ref)
Other Hispanic	1.502	0.803	−2.656^∗∗^	3.614	4.201	1.793
Non‐Hispanic White	0.739	2.345^∗∗∗^	−0.765	−0.439	3.655	−0.488
Non‐Hispanic Black	6.729^∗∗∗^	3.546^∗∗∗^	0.758	2.457	−12.692^∗∗∗^	0.657
Non‐Hispanic Asian	1.025	4.585^∗∗∗^	−1.480^∗^	1.733	12.449^∗∗^	2.741
Other race	0.728	2.755^∗^	−1.810	2.623	−0.232	0.747
Antihypertensive drugs						
Yes	1 (ref)	1 (ref)	1 (ref)	1 (ref)	1 (ref)	1 (ref)
No	1.127	1.526	0.133	13.340^∗∗∗^	−2.860	12.863^∗∗^
Missing	−6.374^∗∗∗^	−3.321^∗∗∗^	−0.929	7.237^∗∗^	−3.818	5.534^∗^
Smoking	0.675	−1.333^∗∗^	−1.767^∗∗^	1.657	10.412^∗∗∗^	1.965
Drinking	−0.262	0.804	3.641^∗∗∗^	−0.687	−2.194	2.537
Diabetes	−0.983	−2.100^∗^	−2.187^∗^	−17.220^∗∗∗^	9.917	−17.397^∗∗∗^

*Note:* Survey‐weighted linear regression.

^∗^ *p* < 0.05.

^∗∗^ *p* < 0.01.

^∗∗∗^ *p* < 0.001 *.*

As shown in Table 3, after adjustment for age, sex, race/ethnicity, smoking, drinking, and diabetes, LDL‐c, triglycerides, and total cholesterol were positively associated with BP. Triglycerides showed significant associations with both SBP and DBP, with *β* coefficients of 0.027 and 0.026, respectively, both *p* < 0.001. LDL‐c was also positively associated with SBP (*β* = 0.026, *p* = 0.015) and DBP (*β* = 0.036, *p* < 0.001), while total cholesterol showed similar positive associations with SBP (*β* = 0.033, *p* = 0.002) and DBP (*β* = 0.036, *p* < 0.001). By contrast, HDL‐c was not significantly associated with SBP (*β* = −0.038, *p* = 0.070), but it showed a significant inverse association with DBP (*β* = −0.059, *p* < 0.001).

**TABLE 3:** Association of serum lipid levels with blood pressure.

Variables	SBP		DBP	
	*β*	*p*	*β*	*p*
HDL cholesterol (mg/dL)	−0.038	0.070	−0.059	< 0.001
LDL cholesterol (mg/dL)	0.026	0.015	0.036	< 0.001
Triglycerides (mg/dL)	0.027	< 0.001	0.026	< 0.001
Total cholesterol (mg/dL)	0.033	0.002	0.036	< 0.001

*Note:* Survey‐weighted linear regression model with age, sex, race, smoking, drinking, and diabetes.

Table 4 shows that adjustment for individual lipid parameters resulted in generally modest attenuation of the association between the TAR and BP, with triglycerides showing the largest reduction across both BMI‐ and WC‐adjusted models. Specifically, triglyceride adjustment reduced the TAR–SBP coefficient by 15.7% in Model 1 and 17.0% in Model 2 and reduced the TAR–DBP coefficient by 15.1% and 16.1%, respectively. By contrast, HDL‐c showed negative attenuation, indicating slight increases in the estimated TAR coefficients after adjustment. LDL‐c was associated with only small attenuation of the TAR–BP association, ranging from 4.3% to 4.4% for SBP and from 5.9% to 6.0% for DBP. Total cholesterol showed slightly greater attenuation than LDL‐c, ranging from 9.2% to 9.8% for SBP and from 8.4% to 9.4% for DBP.

**TABLE 4:** Attenuation of the association between trunk‐to‐appendicular fat ratio and blood pressure after adjustment for serum lipid levels.

Lipid indices		Model 1 (covariates + BMI)			Model 2 (covariates + WC)		
		β (TAR)^a^	β (TAR)^b^	% attenuation	β (TAR)^a^	β (TAR)^b^	% attenuation
SBP	HDL cholesterol (mg/dL)	6.451^∗∗∗^	7.423^∗∗∗^	−15.1	6.007^∗∗∗^	6.765^∗∗∗^	−12.6
	LDL cholesterol (mg/dL)	6.451^∗∗∗^	6.173^∗∗∗^	4.3	6.007^∗∗∗^	5.744^∗∗∗^	4.4
	Triglycerides (mg/dL)	6.451^∗∗∗^	5.436^∗∗∗^	15.7	6.007^∗∗∗^	4.986^∗∗∗^	17.0
	Total cholesterol (mg/dL)	6.451^∗∗∗^	5.858^∗∗∗^	9.2	6.007^∗∗∗^	5.416^∗∗∗^	9.8

DBP	HDL cholesterol (mg/dL)	7.977^∗∗∗^	8.258^∗∗∗^	−3.5	7.185^∗∗∗^	7.440^∗∗∗^	−3.5
	LDL cholesterol (mg/dL)	7.977^∗∗∗^	7.505^∗∗∗^	5.9	7.185^∗∗∗^	6.754^∗∗∗^	6.0
	Triglycerides (mg/dL)	7.977^∗∗∗^	6.772^∗∗∗^	15.1	7.185^∗∗∗^	6.030^∗∗∗^	16.1
	Total cholesterol (mg/dL)	7.977^∗∗∗^	7.304^∗∗∗^	8.4	7.185^∗∗∗^	6.513^∗∗∗^	9.4

^a^Adjusted for age, sex, race/ethnicity, BMI/WC, smoking, drinking, antihypertensive drugs, and diabetes (no lipid included).

^b^Model additionally adjusted for the corresponding lipid parameter.

^∗∗∗^ *p* < 0.001.

## 4. Discussion

In this large, nationally representative study of U.S. adults, we found that a higher TAR is associated with significantly higher BP. TAR demonstrated the strongest correlation with both SBP and DBP among all adiposity measures examined. Each unit increase in TAR corresponded to a substantial rise in SBP and DBP, even after controlling for demographic factors and overall obesity status. Spline analyses further revealed a dose‐dependent relationship—largely linear for SBP and moderately nonlinear for DBP—indicating that BP escalates as TAR increases. We also observed that while serum lipid levels (especially triglycerides) were positively associated with BP, adjusting for these factors attenuated the TAR–BP relationship only modestly. In summary, our results indicate that TAR was a robust independent correlate of BP, and standard lipid parameters accounted for only a modest portion of this association.

A notable finding is that TAR correlated more strongly with BP than did conventional fat measures, including BMI, total body fat percentage, and regional fat mass. In our analysis, the Pearson correlation coefficients between TAR and SBP/DBP (0.32–0.33) exceeded those for trunk fat or appendicular fat alone. This aligns with prior evidence that central adiposity indicators have a stronger association with BP and hypertension risk than general adiposity indicators. BMI often has a weaker correlation with BP, since it fails to capture body fat distribution [2, 20, 21]. Although BMI has been associated with higher cardiovascular risk factors, not every subject who is overweight or obese exhibits alterations in cardiovascular risk factors that are expected from a greater burden of body fat [22]. Our observed TAR–BP correlations (*r* = 0.32–0.33) are comparable to those reported in other cohorts using DXA‐based measures. Kouda et al. noted a similar correlation (*r* = 0.34–0.36) between DXA trunk/limb fat ratio and BP in Japanese adolescents [6], suggesting that the relationship between fat distribution and BP is present across ages and populations. Moreover, children with relatively low overall body fat but a more centrally distributed pattern, as reflected by a higher TAR, have been observed to exhibit higher BP later in life [23]. Collectively, these comparisons suggest that the relative distribution of fat between central and peripheral depots, rather than trunk fat mass alone, may be relevant to BP. This distinction is important because TAR incorporates both trunk fat in the numerator and appendicular fat in the denominator. For a given amount of trunk fat, a lower amount of appendicular fat, particularly lower‐body fat, would produce a higher TAR and may reflect a less favorable central‐to‐peripheral fat pattern. Prior studies have shown that greater proportional leg or gluteofemoral fat is inversely associated with hypertension and several cardiometabolic risk factors, possibly because lower‐body subcutaneous fat may serve as a relatively protective storage depot for excess fatty acids [24–26]. Therefore, the stronger TAR–BP association compared with trunk fat alone may reflect not only greater central fat predominance but also lower relative peripheral fat storage. Because TAR combines arm and leg fat and the present study is cross‐sectional, this interpretation should be considered hypothesis‐generating rather than causal.

A key physiologic finding is that TAR remained associated with BP after adjustment for conventional adiposity measures. In models adjusted for demographic and clinical covariates, and additionally adjusted for either BMI or WC, TAR remained significantly associated with both SBP and DBP, suggesting that TAR captures a distributional component of adiposity not fully represented by general adiposity or waist‐based anthropometric size alone. Moreover, the restricted cubic spline analyses provide additional insight into the shape of this relationship. For SBP, the association with TAR was almost linear across the observed range, with a steady rise in predicted pressure as the ratio increased. For DBP, the curve was flatter at lower values of the ratio and became steeper above roughly 1.0, indicating that the burden of higher diastolic pressure is concentrated in individuals with more pronounced central predominance of fat. This suggests that TAR captures risk information beyond what is provided by BMI and WC alone. In essence, TAR reflects the distributional component of adiposity—particularly central adipose predominance—which confers additional BP risk on top of general adiposity. Our findings align with prior studies, which indicate that DXA‐derived truncal fat proportion is related to cardiometabolic abnormalities independently of total fat mass [6]. Likewise, guidelines from organizations such as the IDF emphasize combining BMI with a measure of central obesity for risk assessment [27], underscoring that distribution conveys unique risk information.

We investigated whether dyslipidemia influences the relationship between central fat distribution and BP. In this analysis, adjustment for lipid parameters resulted in only small reductions in the associations between TAR and BP. The magnitude of the TAR effect remained largely unchanged, suggesting that lipid pathways contribute only a limited portion to this relationship. Although central obesity, characterized by visceral and upper‐trunk fat accumulation, is typically accompanied by adverse lipid profiles, greater fat deposition in the limbs—particularly in the legs—has been shown to exert protective effects [28]. Consequently, the TAR may reflect risk pathways that are only minimally expressed through dyslipidemia. This finding is supported by other research indicating that obesity primarily elevates BP through mechanisms independent of blood lipid levels. Excess visceral fat is known to trigger overactivation of the renin–angiotensin–aldosterone system, leading to renal sodium retention, volume expansion, and increased vascular tone, which in turn raises BP [29, 30]. Abdominal adiposity also provokes systemic inflammation and insulin resistance, which impair endothelial function and vascular compliance [31, 32]. Furthermore, central obesity is associated with sympathetic nervous system overactivity, partly mediated by hyperleptinemia, resulting in increased catecholamine release and elevated heart rate and BP [33–36]. These hemodynamic and neurohormonal effects of visceral fat are thought to be the dominant links between obesity and hypertension [37].

These findings may help clarify the physiologic relevance of central‐to‐peripheral fat patterning in BP variation. They should not be interpreted as evidence that TAR predicts incident hypertension or should be incorporated into routine clinical risk assessment. The present results are more relevant to understanding adiposity‐related BP variation, particularly the contribution of regional fat distribution beyond total adiposity. Longitudinal studies are needed to determine whether TAR predicts future hypertension and whether changes in central‐to‐peripheral fat distribution are accompanied by clinically meaningful changes in BP.

This study has several strengths. It uses a large, nationally representative sample obtained through an established survey design, which supports the generalizability of the findings. Body fat distribution was assessed using DXA, a method that provides accurate and reliable measurements, thereby avoiding the limitations of anthropometric proxies. Multiple adiposity indicators were evaluated within the same framework, using appropriate survey weighting and multivariable adjustment, which allowed for a balanced comparison between TAR and conventional measures. The use of restricted cubic spline modeling further enabled a detailed description of the dose–response pattern between TAR and BP. Exploratory lipid attenuation analysis offered additional insight into whether routinely measured lipid parameters accounted for part of the TAR–BP association. Several limitations should also be acknowledged. The cross‐sectional design prevents causal interpretation, and the direction of association cannot be established. Although DXA performs well in research settings, its cost and limited availability reduce its feasibility for routine clinical use; simpler surrogates for TAR will require further study. Residual confounding cannot be excluded, as some factors, such as dietary sodium or genetic background, were not fully captured. Moreover, we did not evaluate other cardiometabolic biomarkers (e.g., C‐reactive protein, insulin, HOMA‐IR, and leptin), which might further clarify whether additional metabolic pathways account for or confound the TAR–BP relationship. In addition, participants with missing data or those unable to undergo DXA were excluded, which may introduce selection bias. Despite these constraints, this study is among the first to demonstrate in a general adult population that DXA‐derived TAR exhibits a stronger cross‐sectional association with BP than traditional obesity indices, and that standard lipid parameters account for only a modest component of this relationship. These findings support further research on body fat distribution as a physiologic correlate of BP variation.

## 5. Conclusion

In this nationally representative cross‐sectional analysis of US adults, a higher TAR was consistently associated with higher SBP and DBP, even after adjustment for demographic and clinical covariates and either BMI or WC. Restricted cubic spline analyses indicated an almost linear association with SBP and a modestly nonlinear association with DBP, with higher values of the ratio linked to progressively higher pressures. The findings support the concept that central predominance of body fat is an important correlate of BP that is only partly related to lipid abnormalities, although longitudinal studies are needed to determine whether TAR has predictive value for incident hypertension.

## Author Contributions

Conceptualization: Tien Anh Hoang and Bao The Nguyen. Methodology: Tien Anh Hoang and Bao The Nguyen. Data curation: Bao The Nguyen. Formal analysis: Bao The Nguyen. Visualization: Bao The Nguyen. Writing–original draft: Tien Anh Hoang and Bao The Nguyen. Writing–review and editing: Tien Anh Hoang and Bao The Nguyen. Supervision: Tien Anh Hoang. Project administration: Tien Anh Hoang and Bao The Nguyen.

## Funding

This research did not receive any specific grant from funding agencies in the public, commercial, or not‐for‐profit sectors.

## Ethics Statement

NHANES procedures were reviewed and approved by the NCHS Research Ethics Review Board, and all participants provided written informed consent. Because this analysis used publicly available, de‐identified NHANES data, no additional institutional ethics approval was required.

## Consent

Please see the Ethics Statement.

## Conflicts of Interest

The authors declare no conflicts of interest.

## Supporting Information

Additional supporting information can be found online in the Supporting Information section.

## Supporting information


**Supporting Information** Supporting Tables. The supporting material for this study includes five supporting tables. Supporting Table S1 presents the comparison of survey‐weighted correlations of trunk‐to‐appendicular fat ratio and waist‐to‐height ratio with systolic and diastolic blood pressure. Supporting Table S2 presents the incremental explanatory value of trunk‐to‐appendicular fat ratio beyond total fat mass for blood pressure. Supporting Table S3 presents the sex‐specific association between trunk‐to‐appendicular fat ratio and blood pressure in survey‐weighted interaction models. Supporting Table S4 presents the race/ethnicity‐specific association between trunk‐to‐appendicular fat ratio and systolic blood pressure. Supporting Table S5 presents pairwise comparisons of race/ethnicity‐specific trunk‐to‐appendicular fat ratio–systolic blood pressure slopes.

## Data Availability

All data used in this study are publicly available from the NHANES database of the U.S. Centers for Disease Control and Prevention (CDC) at https://www.cdc.gov/nchs/nhanes/. No additional datasets beyond NHANES were generated or analyzed.

## References

[1] Chen L. , Zhang J. , Zhou N. , Weng J.-Y. , Bao Z.-Y. , and Wu L.-D. , Association of Different Obesity Patterns With Hypertension in US Male Adults: A Cross-Sectional Study, Scientific Reports. (2023) 13, no. 1, 10.1038/s41598-023-37302-x. PMC10310720 37386040

[2] Moges B. , Amare B. , Fantahun B. , and Kassu A. , High Prevalence of Overweight, Obesity, and Hypertension With Increased Risk to Cardiovascular Disorders Among Adults in Northwest Ethiopia: A Cross Sectional Study, BMC Cardiovascular Disorders. (2014) 14, no. 1, 10.1186/1471-2261-14-155. PMC4228065 25373922

[3] Santulli G. , Epidemiology of Cardiovascular Disease in the 21st Century: Updated Numbers and Updated Facts, Journal of Cardiovascular Disease. (2013) 1, no. 1, 1–2.

[4] Ren H. , Guo Y. , Wang D. , Kang X. , and Yuan G. , Association of Normal-Weight Central Obesity With Hypertension: A Cross-Sectional Study From the China Health and Nutrition Survey, BMC Cardiovascular Disorders. (2023) 23, no. 1, 10.1186/s12872-023-03126-w. PMC9996911 36890477

[5] Pluta W. , Lubkowska A. , and Dudzińska W. , Diagnostic and Prognostic Value of Adipose Tissue Content and Distribution Indicators for Normal Weight Obesity in Young Women, Scientific Reports. (2025) 15, no. 1, 10.1038/s41598-025-12262-6. PMC12485104 41028841

[6] Kouda K. , Fujita Y. , Ohara K. et al., Associations Between Trunk-to-Peripheral Fat Ratio and Cardiometabolic Risk Factors in Elderly Japanese Men: Baseline Data From the Fujiwara-Kyo Osteoporosis Risk in Men (FORMEN) Study, Environmental Health and Preventive Medicine. (2021) 26, no. 1, 10.1186/s12199-021-00959-9. PMC7980554 33743595

[7] Bays H. E. , Kirkpatrick C. F. , Maki K. C. et al., Obesity, Dyslipidemia, and Cardiovascular Disease: A Joint Expert Review From the Obesity Medicine Association and the National Lipid Association 2024, Journal of Clinical Lipidology. (2024) 18, no. 3, e320–e350, 10.1016/j.jacl.2024.04.001. 38664184

[8] Dąbrowska E. and Narkiewicz K. , Hypertension and Dyslipidemia: The Two Partners in Endothelium-Related Crime, Current Atherosclerosis Reports. (2023) 25, no. 9, 605–612, 10.1007/s11883-023-01132-z. 37594602 PMC10471742

[9] Gallo G. , Volpe M. , and Savoia C. , Endothelial Dysfunction in Hypertension: Current Concepts and Clinical Implications, Frontiers of Medicine. (2021) 8, 10.3389/fmed.2021.798958. PMC8811286 35127755

[10] Parvanova A. , Reseghetti E. , Abbate M. , and Ruggenenti P. , Mechanisms and Treatment of Obesity-Related Hypertension-Part 1: Mechanisms, Clinical Kidney Journal. (2024) 17, no. 1, 10.1093/ckj/sfad282. PMC10768772 38186879

[11] Kouda K. , Nakamura H. , Fujita Y. , Ohara K. , and Iki M. , Increased Ratio of Trunk to Appendicular Fat and Increased Blood Pressure: Study of a General Population of Hamamatsu Children, Circulation Journal. (2012) 76, no. 12, 2848–2854, 10.1253/circj.cj-12-0417. 22893277

[12] Centers for Disease Control and Prevention (CDC) , National Center for Health Statistics (NCHS), 2025, National Health and Nutrition Examination Survey, https://wwwn.cdc.gov/nchs/nhanes/.

[13] R Core Team , A Language and Environment for Statistical Computing, Foundation for Statistical Computing, https://www.R-project.org/.

[14] Lumley T. , Complex Surveys: A Guide to Analysis Using R, 2011, John Wiley and Sons.

[15] Harrell F. E. , Multivariable Modeling Strategies, Regression Modeling Strategies: With Applications to Linear Models, Logistic and Ordinal Regression and Survival Analysis. (2015) 63–102.

[16] Wickham H. and Data Analysis , Ggplot2: Elegant Graphics for Data Analysis, 2016, Springer, 189–201.

[17] Tong T. Y. N. , Imamura F. , Monsivais P. et al., Dietary Cost Associated With Adherence to the Mediterranean Diet, and its Variation by Socio-Economic Factors in the UK Fenland Study, British Journal of Nutrition. (2018) 119, no. 6, 685–694, 10.1017/s0007114517003993. 29553031 PMC5999016

[18] Lee P. H. , Is a Cutoff of 10% Appropriate for the Change-in-Estimate Criterion of Confounder Identification?, Journal of Epidemiology. (2014) 24, no. 2, 161–167, 10.2188/jea.je20130062. 24317343 PMC3983286

[19] VanderWeele T. J. , Principles of Confounder Selection, European Journal of Epidemiology. (2019) 34, no. 3, 211–219, 10.1007/s10654-019-00494-6. 30840181 PMC6447501

[20] Lee C. M. Y. , Huxley R. R. , Wildman R. P. , and Woodward M. , Indices of Abdominal Obesity are Better Discriminators of Cardiovascular Risk Factors than BMI: A Meta-Analysis, Journal of Clinical Epidemiology. (2008) 61, no. 7, 646–653, 10.1016/j.jclinepi.2007.08.012. 18359190

[21] Huynh M. V. , Le Thi H. T. , Tran N. T. , Hoang T. Y. B. , and Hoang A. T. , The Relationship Between Body Mass Index and Blood Pressure in Vietnam, Iranian Heart Journal. (2025) 26, no. 1, 6–15.

[22] Piché M. E. , Tchernof A. , and Després J. P. , Obesity Phenotypes, Diabetes, and Cardiovascular Diseases, Circulation Research. (2020) 126, no. 11, 1477–1500, 10.1161/circresaha.120.316101. 32437302

[23] Kouda K. , Ohara K. , Fujita Y. , Nakamura H. , and Iki M. , Trunk-to-Peripheral Fat Ratio Predicts Subsequent Blood Pressure Levels in Pubertal Children With Relatively Low Body Fat-Three-Year Follow-up Study, Circulation Journal. (2016) 80, no. 8, 1838–1845, 10.1253/circj.cj-16-0259. 27295997

[24] Visaria A. , Lo D. , Maniar P. , Dave B. , and Joshi P. , Leg and Arm Adiposity is Inversely Associated With Diastolic Hypertension in Young and Middle-Aged United States Adults, Clinical Hypertension. (2022) 28, no. 1, 10.1186/s40885-021-00190-2. PMC8760692 35031064

[25] Manolopoulos K. N. , Karpe F. , and Frayn K. N. , Gluteofemoral Body Fat as a Determinant of Metabolic Health, International Journal of Obesity. (2010) 34, no. 6, 949–959, 10.1038/ijo.2009.286. 20065965

[26] Alser M. , Naja K. , and Elrayess M. A. , Mechanisms of Body Fat Distribution and Gluteal-Femoral Fat Protection Against Metabolic Disorders, Frontiers in Nutrition. (2024) 11, 10.3389/fnut.2024.1368966. PMC10999599 38590830

[27] Quah Y. V. Jr. , Poh B. K. , and Ismail M. N. , Metabolic Syndrome Based on IDF Criteria in a Sample of Normal Weight and Obese School Children, Malaysian Journal of Nutrition. (2010) 16, no. 2, 207–217. 22691926

[28] Feingold K. R. , Feingold K. R. , Ahmed S. F. , Anawalt B. et al., Obesity and Dyslipidemia, Endotext. MDText.com, Inc. Copyright © 2000–2025, 2000, MDText.com, Inc.

[29] Srinivasa S. , Fitch K. V. , Wong K. et al., RAAS Activation Is Associated With Visceral Adiposity and Insulin Resistance Among HIV-Infected Patients, Journal of Clinical Endocrinology and Metabolism. (2015) 100, no. 8, 2873–2882, 10.1210/jc.2015-1461. 26086328 PMC4525005

[30] Goswami B. , Reang T. , Sarkar S. , Sengupta S. , and Bhattacharjee B. , Role of Body Visceral Fat in Hypertension and Dyslipidemia Among the Diabetic and Nondiabetic Ethnic Population of Tripura-A Comparative Study, Journal of Family Medicine and Primary Care. (2020) 9, no. 6, 2885–2890, 10.4103/jfmpc.jfmpc_187_20. PMC7491841 32984144

[31] Yoo J. K. and Fu Q. , Impact of Sex and Age on Metabolism, Sympathetic Activity, and Hypertension, FASEB Journal. (2020) 34, no. 9, 11337–11346, 10.1096/fj.202001006rr. 32779294

[32] Lyon C. J. , Law R. E. , and Hsueh W. A. , Minireview: Adiposity, Inflammation, and Atherogenesis, Endocrinology. (2003) 144, no. 6, 2195–2200, 10.1210/en.2003-0285. 12746274

[33] Gruber T. , Pan C. , Contreras R. E. et al., Obesity-Associated Hyperleptinemia Alters the Gliovascular Interface of the Hypothalamus to Promote Hypertension, Cell Metabolism. (2021) 33, no. 6, 1155–1170.e10, 10.1016/j.cmet.2021.04.007. 33951475 PMC8183500

[34] Engeli S. and Sharma A. M. , The Renin-Angiotensin System and Natriuretic Peptides in Obesity-Associated Hypertension, Journal of Molecular Medicine (Berlin). (2001) 79, no. 1, 21–29, 10.1007/s001090000144. 11327100

[35] Hall J. E. , The Kidney, Hypertension, and Obesity, Hypertension. (2003) 41, no. 3 Pt 2, 625–633, 10.1161/01.hyp.0000052314.95497.78. 12623970

[36] Hall M. E. , do Carmo J. M. , da Silva A. A. , Juncos L. A. , Wang Z. , and Hall J. E. , Obesity, Hypertension, and Chronic Kidney Disease, International Journal of Nephrology and Renovascular Disease. (2014) 7, 75–88, 10.2147/ijnrd.s39739. 24600241 PMC3933708

[37] Hall J. E. , do Carmo J. M. , da Silva A. A. , Wang Z. , and Hall M. E. , Obesity-Induced Hypertension, Circulation Research. (2015) 116, no. 6, 991–1006, 10.1161/circresaha.116.305697. 25767285 PMC4363087

