# Supplementary material for: Trunk‐to‐Appendicular Fat Ratio and Blood Pressure: Survey‐Weighted Regression, Spline Modeling, and Exploratory Lipid Attenuation Analysis
Source: Int J Hypertens. 2026 Jul 18;2026:3281602. doi: 10.1155/ijhy/3281602 (PMC13379948; doi:10.1155/ijhy/3281602)
Supplement: Supplementary file 1 — Supporting Information Supporting Tables. The supporting material for this study includes five supporting tables. Supporting Table S1 presents the comparison of survey‐weighted correlations of trunk‐to‐appendicular fat ratio and waist‐to‐height ratio with systolic and diastolic blood pressure. Supporting Table S2 presents the incremental explanatory value of trunk‐to‐appendicular fat ratio beyond total fat mass for blood pressure. Supporting Table S3 presents the sex‐specific association between trunk‐to‐appendicular fat ratio and blood pressure in survey‐weighted interaction models. Supporting Table S4 presents the race/ethnicity‐specific association between trunk‐to‐appendicular fat ratio and systolic blood pressure. Supporting Table S5 presents pairwise comparisons of race/ethnicity‐specific trunk‐to‐appendicular fat ratio–systolic blood pressure slopes. [file IJHY-2026-3281602-s001.docx]

**Supplementary Table S1. Comparison of survey-weighted correlations of TAR and WHtR with blood pressure**

| **BP** | **r TAR** | **r WHtR** | **∆ r** | **95%CI** | **p** |
| --- | --- | --- | --- | --- | --- |
| SBP | 0.326 | 0.269 | 0.058 | 0.023 - 0.092 | 0.001 |
| DBP | 0.330 | 0.233 | 0.097 | 0.060 - 0.134 | <0.001 |

**Supplementary Table S2. Incremental explanatory value of TAR beyond total fat mass for blood pressure**

| **BP** | **Base model R² with total fat mass** | **R² after adding TAR** | **ΔR²** | **Additional variance explained** | **p for added TAR** |
| --- | --- | --- | --- | --- | --- |
| SBP | 0.224 | 0.236 | 0.0116 | 0.0116 | <0.001 |
| DBP | 0.169 | 0.190 | 0.0207 | 0.0207 | <0.001 |

**Supplemetary Table S3. Sex-specific association between trunk-to-appendicular fat ratio and blood pressure in survey-weighted interaction models**

| **Outcome** | **p interaction TAR × sex** | **TAR slope in men** | **TAR slope in women** |
| --- | --- | --- | --- |
| SBP | 0.002 | 4.28, 95% CI 0.79–7.77 | 11.1, 95% CI 7.50–14.6 |
| DBP | 0.031 | 8.75, 95% CI 5.60–11.9 | 5.07, 95% CI 2.44–7.71 |

**Supplementary Table S4. Race/ethnicity-specific association between trunk-to-appendicular fat ratio and systolic blood pressure**

| **Race/ethnicity** | **TAR–SBP slope, β** | **SE** | **95% CI** | **p** |
| --- | --- | --- | --- | --- |
| Mexican American | 5.973 | 2.020 | 1.933 to 10.014 | 0.004 |
| Other Hispanic | 14.010 | 3.670 | 6.669 to 21.351 | <0.001 |
| Non-Hispanic White | 5.958 | 1.740 | 2.474 to 9.442 | 0.001 |
| Non-Hispanic Black | 17.862 | 3.380 | 11.095 to 24.630 | <0.001 |
| Non-Hispanic Asian | 10.891 | 2.930 | 5.027 to 16.755 | <0.001 |
| Other race | -2.622 | 3.190 | -8.994 to 3.749 | 0.414 |

**Supplementary Table S5. Pairwise comparisons of race/ethnicity-specific TAR–SBP slopes**

| **Contrast** | **Difference in**  **TAR–SBP slope** | **95% CI** | **p** | **Holm-adjusted p** |
| --- | --- | --- | --- | --- |
| Non-Hispanic White vs Non-Hispanic Black | -11.904 | -17.968 to -5.840 | <0.001 | 0.003 |
| Mexican American vs Non-Hispanic Black | -11.889 | -19.787 to -3.991 | 0.004 | 0.042 |
| Other Hispanic vs Other race | 16.632 | 7.080 to 26.184 | <0.001 | 0.012 |
| Non-Hispanic Black vs Other race | 20.484 | 12.260 to 28.708 | <0.001 | <0.001 |
| Non-Hispanic Asian vs Other race | 13.513 | 5.365 to 21.661 | 0.002 | 0.018 |
